# Comparison of swept-source OCTA and indocyanine green angiography in central serous chorioretinopathy

**DOI:** 10.1186/s12886-022-02607-4

**Published:** 2022-09-22

**Authors:** Qiaozhu Zeng, Yuou Yao, Siying Li, Zhi Yang, Jinfeng Qu, Mingwei Zhao

**Affiliations:** 1 Department of Ophthalmology, Eye Diseases and Optometry Institute, Peking University People’s Hospital, No. 11 Xizhimen South Street, Xicheng District, Beijing, China; 2 Beijing Key Laboratory of Diagnosis and Therapy of Retinal and Choroid Diseases, College of Optometry, Peking University Health Science Center, Beijing, China; 3 TowardPi (Beijing) Medical Technology, Beijing, China

**Keywords:** Swept-source optical coherence tomography angiography, Indocyanine green angiography, Central serous chorioretinopathy, Jaccard index

## Abstract

**Background:**

To compare swept-source optical coherence tomography angiography (SS-OCTA) and indocyanine green angiography (ICGA) in patients with central serous chorioretinopathy (CSC).

**Methods:**

SS-OCTA and ICGA images of 39 eyes with symptomatic CSC were collected and aligned. Spatial overlap of the annotations of the coarse granulated high reflective area on choriocapillary OCTA and the hyperfluorescence area on mid-phase ICGA was calculated according to the Jaccard index (JI). SS-OCTA findings of fellow eyes and changes in SS-OCTA abnormalities during the follow-up were also analyzed.

**Results:**

Three main types of abnormalities in choriocapillaris SS-OCTA images were found: type A, coarse granulated high reflective area (39 eyes [100%]); type B, roundish dark halo around Type A (32 eyes [82.1%]); and type C, coarse granulated low reflective area (39 eyes [100%]). The mean JI of type A on SS-OCTA and the hyperfluorescence area on ICGA were 0.55 ± 0.15 for grader 1 and 0.49 ± 0.15 for grader 2. The mean area of type A abnormalities on SS-OCTA and hyperfluorescence on ICGA was 3.976 (IQR, 2.139–8.168) and 3.043 (IQR, 1.408–4.909) mm^2^ (*P* = 0.199). The areas of type A, B and C abnormalities on SS-OCTA after laser treatment or observation were 3.36mm^2^ (IQR, 2.399–9.312), 2.9mm^2^ (IQR, 2.15–3.7), and 0.19mm^2^ (IQR, 0.08–0.23), respectively, which was smaller than those in the baseline (7.311mm^2^ (IQR 3.788–11.209), *P* < 0.001; 4.3mm^2^ (IQR, 2.8–9.8), *P* = 0.002;0.33mm^2^ (IQR, 0.23–0.38), *P* < 0.001). The change in the type A, B or C area was not significantly different between the two groups (*P* = 0.679, 0.732, and 0.892).

**Conclusion:**

The coarse granulated high reflective area in SS-OCTA corresponded well with the hyperpermeability area in ICGA. SS-OCTA promotes noninvasive visualization and follow-up quantifications of the choroidal vasculature in CSC patients.

**Supplementary Information:**

The online version contains supplementary material available at 10.1186/s12886-022-02607-4.

## Background

Central serous chorioretinopathy (CSC) is a chorioretinal disease characterized by serous retinal detachment with or without retinal pigment epithelium (RPE) detachment. CSC occurs 6 times more commonly in males than in females, with an average age at onset of 39 to 51 years [1]. It is clinically characterized by diminution of central vision, metamorphopsia and central scotoma. Most acute CSCs with a disease duration less than 6 months are self-limited, while 30%-50% of acute CSCs may result in permanent vision impairment and recurrence within 1 year [2]. Chronic CSC is associated with multifocal leakage on fluorescein angiography (FA), widespread RPE alterations and photoreceptor defects, leading to progressive vision loss [3]. Abnormality in choroidal vasculature has been an established factor for the pathogenesis of CSC, including the dilatation and hyperpermeability of choroidal vessels resulting in the accumulation of subretinal fluid (SRF).

Advanced imaging technologies during the last few decades have improved our understanding of CSC. Choriocapillary congestion, increased choroidal permeability, leakage into interstitial or stromal space, and dilatation of choroidal vessels are depicted on indocyanine green angiogram (ICGA) [4–7]. However, ICGA is an invasive technique that requires trained technicians and patient cooperation. In addition, the reproducible quantification of lesions for the differential diagnosis and follow-up is not possible using ICGA.

Optical coherence tomography angiography (OCTA) is a quantitative, noninvasive, and dye-free technique that allows for the imaging of different layers [8, 9]. Swept-source OCTA (SS-OCTA) has been extensively investigated since its first report in 2006 [10]. An SS-OCTA device is available from TowardPi Medical Technology (TowardPi Medical Technology Co., Ltd, Beijing, China): BM400K BMizar. With the combination of a long wavelength (1060 nm) full range swept source and 400 kHz A-scan rate, the device has the capability to acquire as deep as 6 mm scan depth and high resolution. The algorithm to detect motion signals is called higher-order moments amplitude decorrelation angiography (HMADA). It is an effective and proprietary visualization technique of the capillary network in the choroidal circulations, by capturing higher order statistical signals in OCTA data. It could provide additional information on the blood supply in CSC and therefore help us better understand the pathophysiology of the disease. Herein, we aim to describe SS-OCTA findings in patients with CSC and to compare SS-OCTA with traditional ICGA, as well as describe the area change of abnormalities on SS-OCTA after treatment.

## Methods

### Patients

Patients with symptomatic CSC were consecutively included in this study from August 2021 to October 2021 at the Department of Ophthalmology, Peking University People’s Hospital. Acute CSC is defined as an acute-onset, dome-shaped serous detachment of the neuroretina, with spontaneous complete resolution of the SRF in 6 months and a good visual prognosis. Chronic CSC was diagnosed based on the duration of persistent fluid for at least 6 months, as well as multifocal leakage, widespread RPE alterations and photoreceptor alterations [11].

The inclusion criteria were as follows: 1) presence of SRF involving the fovea with or without PED on OCT, 2) evidence of active leakage on FA, 3) abnormal dilated choroidal vasculature on ICGA, and 4) no previous treatments. Patients were excluded if: 1) any other ocular diseases associated with SRF, such as choroidal neovascularization (CNV), polypoidal choroidal vasculopathy (PCV), diabetic retinopathy (DR), retinal vein occlusion (RVO), Coats’ disease, etc. 2) any disease that may affect the quality of imaging (quality of OCT or OCTA < 7), such as cataract, high myopia, or nystagmus; 3) severe kidney or liver dysfunction and/or unstable cardiac disease; 4) pregnancy; and 5) any conditions rendering patients intolerable to image acquisitions.

All included patients underwent a full ophthalmic examination, including measurement of best corrected distance visual acuity (BCVA), intraocular pressure (IOP), axial length (AL), spherical equivalent, slit lamp examination, and indirect ophthalmoscopy. Data on baseline demographics (sex, age, type of CSC, history of hypertension, history of diabetes mellitus, etc.) and current ophthalmologic examination findings were collected.

Our study adhered to the tenets of the Declaration of Helsinki. Informed consent was obtained from all patients, and the protocol was approved by the Ethics Committee of People’s Hospital of Peking University.

### Image acquisition

Patients were imaged with a 400 kHz SS-OCTA instrument (BM400K BMizar, TowardPi Medical Technology Co., Ltd, Beijing, China). With artificial intelligence (AI) technology, each layer, including Bruch’s membrane (BM) and choroid-sclera interface, can be recognized. We manually verified the accuracy of automatic segmentation with B-scans. The slab used for comparison with ICGA was the choriocapillaris layer. ICGA was obtained from the Spectralis HRA + OCT device (Heidelberg Engineering, Heidelberg, Germany). All OCTA examinations were performed by one well-trained physician (ZQZ) on the same day when ICGA was performed. Choriocapillary OCTA images and representative ICGA images at early (within 30 s postinjection) and mid phase (60–180 s postinjection) were exported in TIF format.

### Image processing

**Fig. 1:**
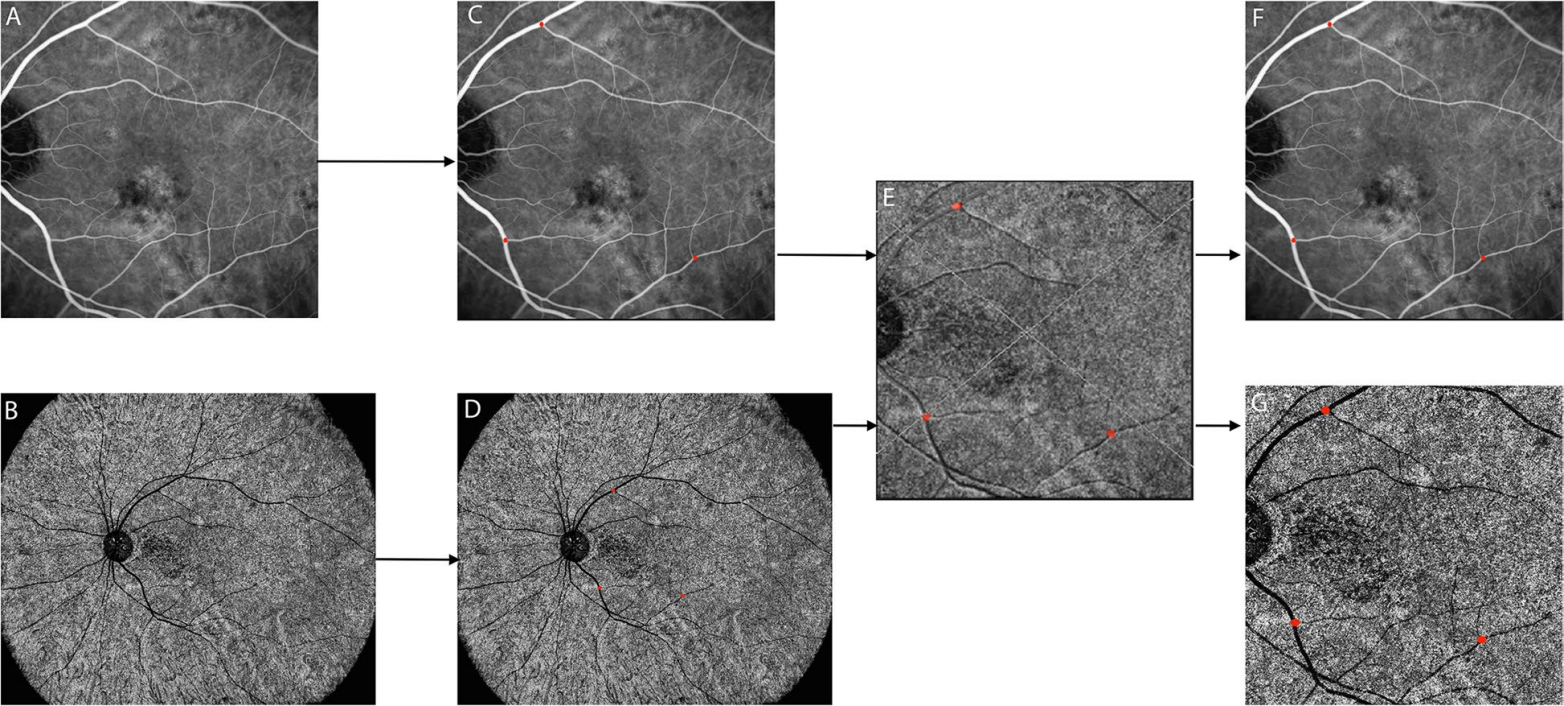
A representative example of image registration. With the method of translation, rotation, and rescaling the target images, three user-specified landmarks on both SS-OCTA and ICGA **A-E** were accurately matched. After the registration, two same size images **F**,** G** from the two modalities were obtained for further comparison and Jaccard index measurement

We aligned the OCTA images and ICGA images using Photoshop CS6 (version 13.0.0.0, Adobe, San Jose, CA, USA). After translation, rotation, and rescaling of the target images, three user-specified landmarks on both modalities were matched. A representative example of registered images is shown in Fig. 1. Two experienced and masked observers (QJF, ZMW) separately read and annotated abnormal areas on choriocapillary OCTA images, hyperfluorescence areas on mid-phase ICGA images, and hypofluorescence areas on early-phase ICGA images. The annotation was performed by the region-of-interest (ROI) function of ImageJ (bundled with Java 1.8.0_172).

### Image analysis

**Fig. 2:**
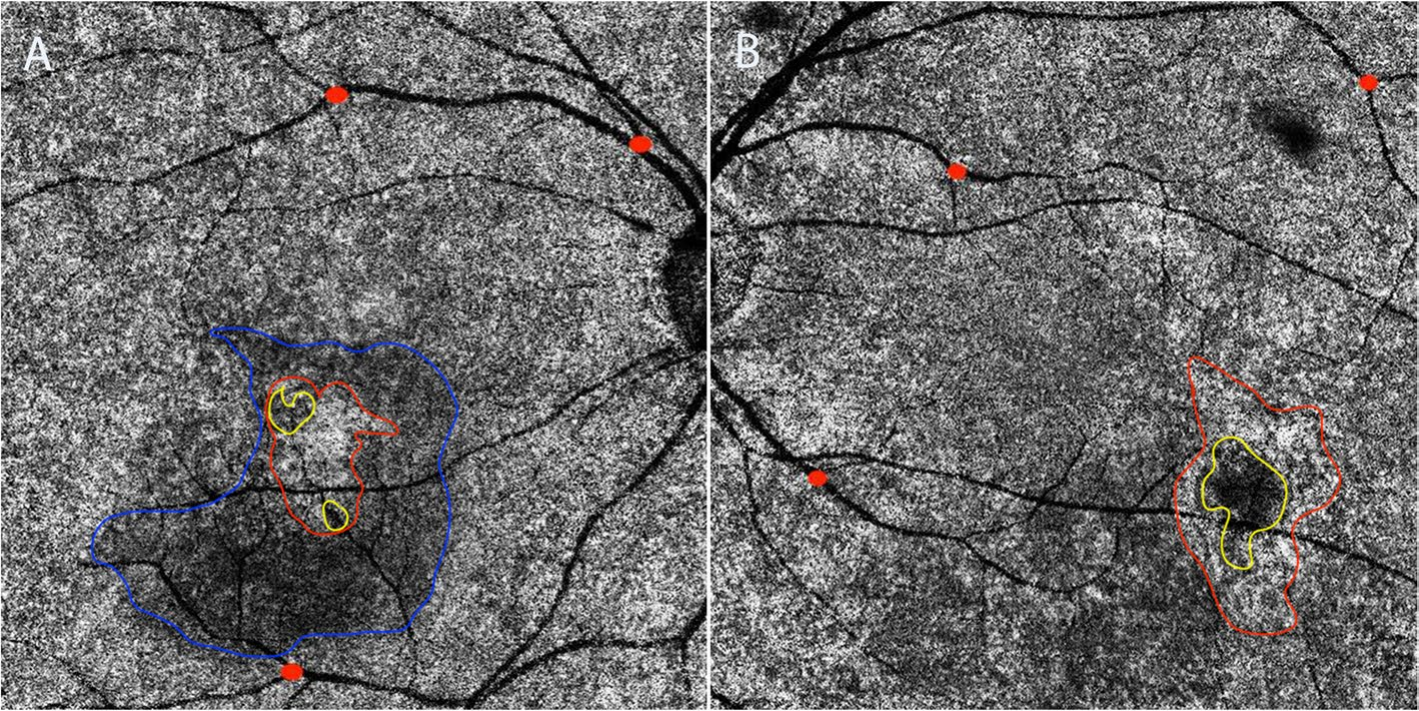
Three main types of abnormalities were found in choriocapillaris layer in SS-OCTA. Type A (outlined in red), was defined as coarse granulated high reflective area; type B (outlined in blue) was defined as the roundish dark halo around type A; and type C (outlined in yellow) was defined as the coarse granulated low reflective spot inside type A. The left image **A** indicates a representative case with all the three types of abnormalities on choriocapillaris layer. The right panel **B** represent for a case without type B abnormality

From the choriocapillary OCTA images, we identified three types of abnormalities [12]: type A, coarse granulated high reflective area (outlined in red in Fig. 2); type B, roundish dark halo around type A (outlined in blue in Fig. 2); and type C, coarse granulated low reflective area (outlined in yellow in Fig. 2).

To determine the agreement of annotations between the ICGA and SS-OCTA images, the spatial overlap of the annotations of the coarse granulated high reflective area (type A) on choriocapillary OCTA and the hyperfluorescence area on mid-phase ICGA was calculated separately according to the Jaccard index (JI) as follows:

$$JI = \frac{|A\cap B|}{|A\cup B|}=\mathrm{IA}\cap \mathrm{BI}/(\mathrm{IAI}+\mathrm{IBI}-\mathrm{IA}\cap \mathrm{BI})$$

**Fig. 3:**
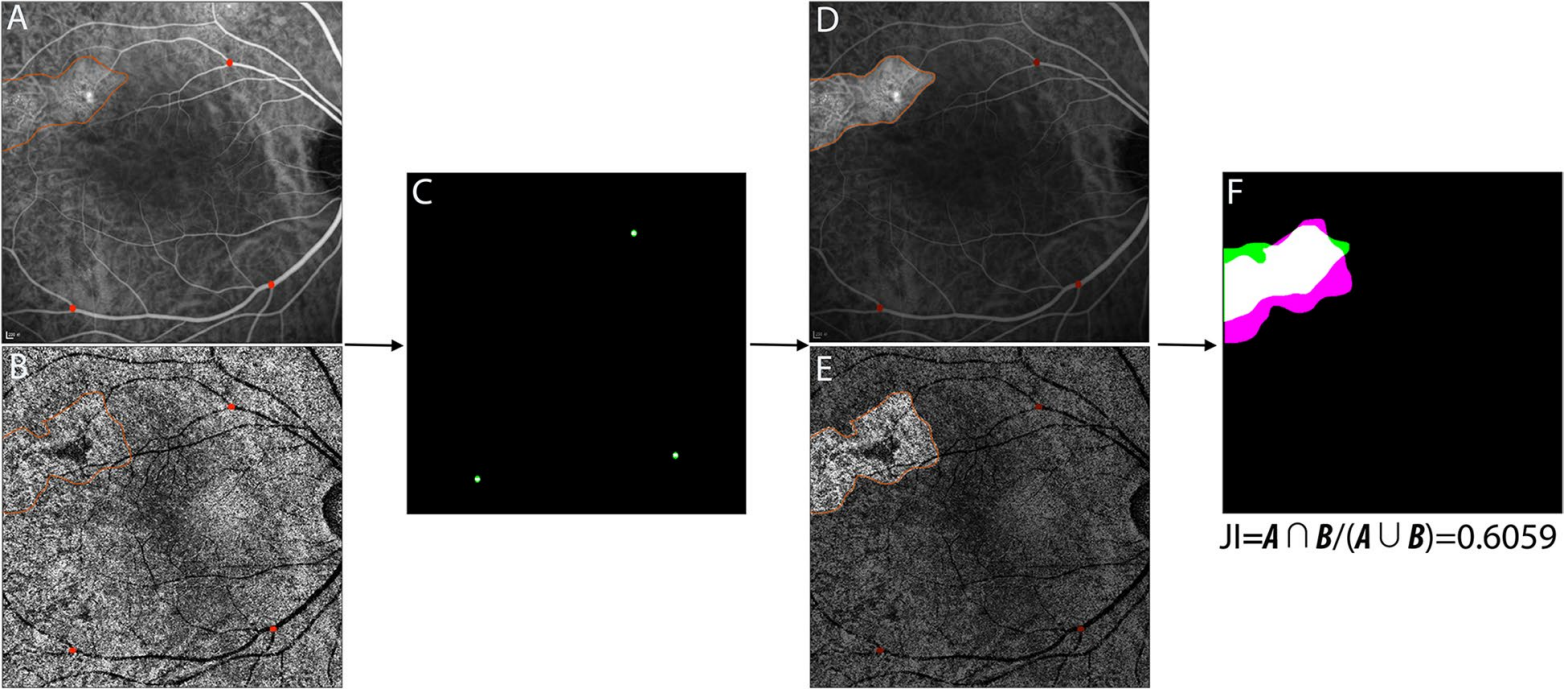
A representative example of Jaccard index (JI) calculation. Two annotations of region-of-interest were performed on two images using ImageJ **A**, **B**; It was verified that three user-specified landmarks on both SS-OCTA and ICGA were accurately matched **C**; The annotations of ROI on both images were extracted **D**, **E**; The two images were overlapped and the areas of intersection and union were calculated using affine transformation **F**; JI = (|A ∩ B|)/(|A ∪ B|) = 0.6059

Similar to our previous study [12], A and B represent the area covering the annotation of ICGA and choriocapillary OCTA. JI was defined as the ratio of intersection area of the two ROIs divided by the union area of the ROI. A representative example of how they were defined is shown in Fig. 3. The interobserver agreement of image annotations between the two graders was measured in the same manner according to JI. A model of affine transformation was applied in imaging analysis:

$$\left[\begin{array}{c}u\\ v\end{array}\right] = \left[\begin{array}{ccc}{a}_{00}& {a}_{01}& {a}_{02}\\ {a}_{10}& {a}_{11}& {a}_{12}\end{array}\right]\left[\begin{array}{c}x\\ y\\ 1\end{array}\right]$$

The area of type A and C abnormalities on OCTA and the hyperfluorescence area on ICGA were finally decided after adjudication between the two graders. All patients were followed up for at least 1 month. The area changes of abnormalities on SS-OCTA were also calculated.

### Statistical analysis

All statistical analyses were performed with Stata/SE 15.0 (V.15.0; Stata, College Station, TX, USA). For patient characteristics, descriptive methods, with standard summary statistics including the mean (S.D., standard deviation), median, interquartile range (IQR), and proportions were applied. For paired comparisons between JIs, Student’s t test (t test) was performed to compare normally distributed quantitative variables, while the nonparametric Wilcoxon signed rank test was used for nonnormally distributed quantitative variables. Two-way analysis of variance (ANOVA) was used to compare the median change in the target area between two groups. *P* < 0.05 was considered to be statistically significant.

## Results

### Demographics

Patient characteristics were depicted in Table 1. Thirty-nine eyes of 34 patients (8 women and 26 men) were included in this study, with a mean age of 46.8 ± 7.8 years. There were 22 (56.4%) and 17 (43.6%) eyes diagnosed with acute and chronic CSC, respectively. Seven (20.6%) patients had hypertension and 1 (2.9%) patient had diabetes mellitus. Twenty-nine (85.3%) patients presented with unilateral involvement, while the disease occurred bilaterally in 5 (14.7%) patients. The median BCVA was 0.1 (range, 0.02–0.18) logMAR (logarithmic minimum angle of resolution). The median IOP was 14.4 (range, 11.8–16) mmHg. The median AL of all the included eyes was 23.9 (range, 23–24.6 mm. The mean spherical diopter was -0.7 (2.2) D.

**Table 1:** Demographic and clinical characteristics of the patients included in this study

Parameters	Population (*n* = 34)
Eyes involved, n (%)	
Unilateral	29 (85.3)
Bilateral	5 (14.7)
Age, years, mean (± SD)	46.8 (7.8)
Male: female	13:4
Hypertension, n (%)	7 (20.6)
Diabetes mellitus, n (%)	1 (2.9)
	Eyes (*n* = 39)
Eyes involved, n (%)	
Right	20 (51.3)
Left	19 (48.7)
BCVA, logMAR, median (IQR)	0.1 (0.02, 0.18)
IOP, mmHg, median (IQR)	14.4 (11.8, 16)
AL, mm, median (IQR)	23.9 (23.0, 24.6)
Spherical diopter, D, mean (± SD)	-0.7 (2.2)
Type of CSC, n (%)	
acute	22 (56.4)
chronic	17 (43.6)

*SD* Standard deviation, *BCVA* Best corrected distance visual acuity, *logMAR* Logarithmic minimum angle of resolution, *IQR* Interquartile range, *IOP* Intraocular pressure, *AL* Axil length, *CSC* Central serous chorioretinopathy

### ICGA and SS-OCTA findings in CSC eyes

**Fig. 4:**
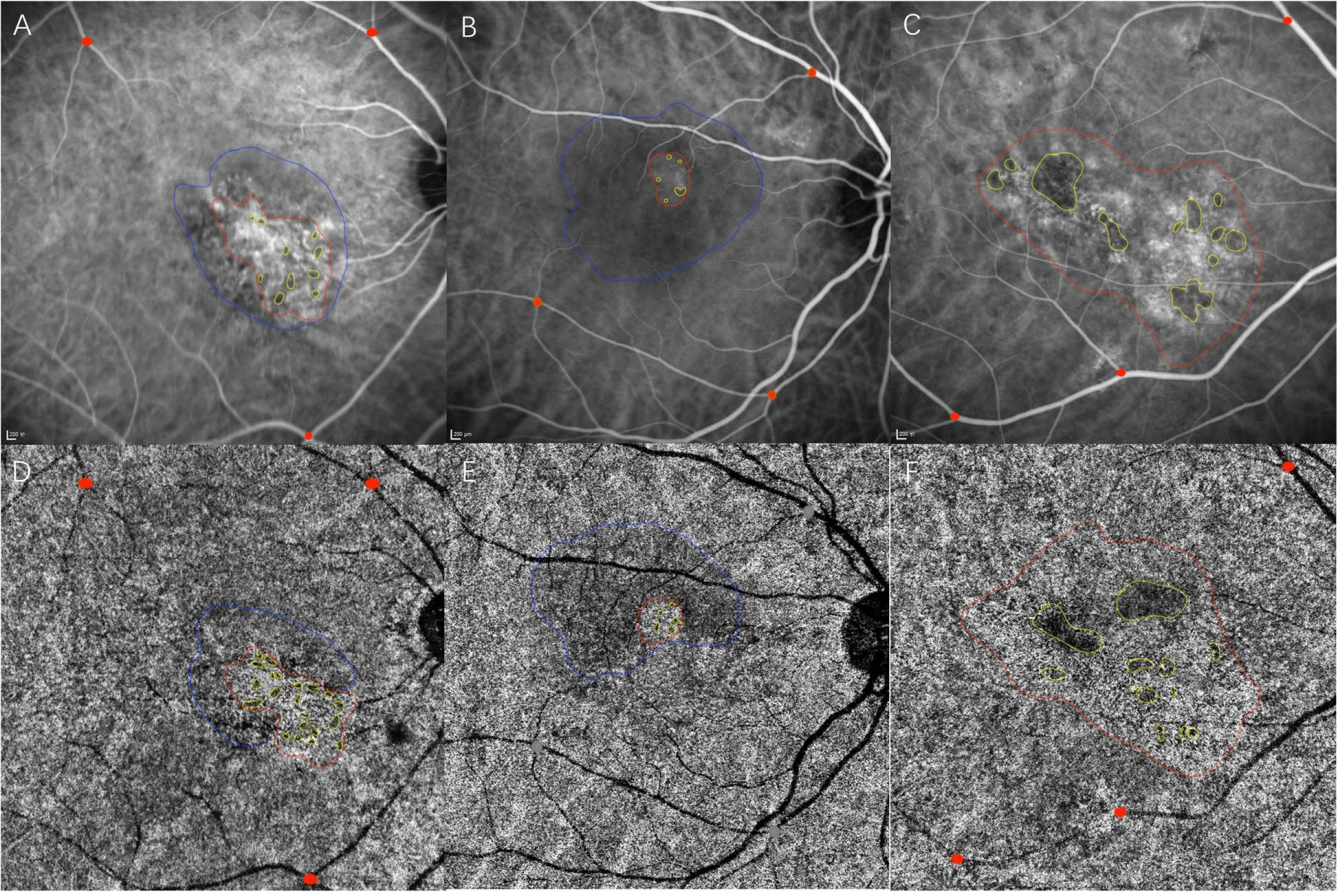
Examples of type A, B and C abnormalities in SS-OCTA and corresponding areas in ICGA **A-D**

Examples of type A, B and C abnormalities in SS-OCTA and corresponding areas in ICGA were shown in Fig. 4. In ICGA, choroidal hyperpermeability was seen in all the 39 eyes of patients, including hypofluorescence area inside the hyperpermeability in 32 (82.1%) eyes and a hypofluorescence halo around the hyperpermeability in 20 (51.3%) eyes. Clear dye leakage in 20 (51.3%) eyes was seen in the late stage of ICGA. We observed abnormal choriocapillaris in SS-OCTA in all the 39 eyes. Type A, B and C abnormalities were found in 39 (100%), 32 (82.1%) and 39 (100%) eyes, respectively. Twenty-two (82.1%) eyes exhibited all three types of abnormalities. Seven (17.9%) had type A and C abnormalities.

**Fig. 5:**
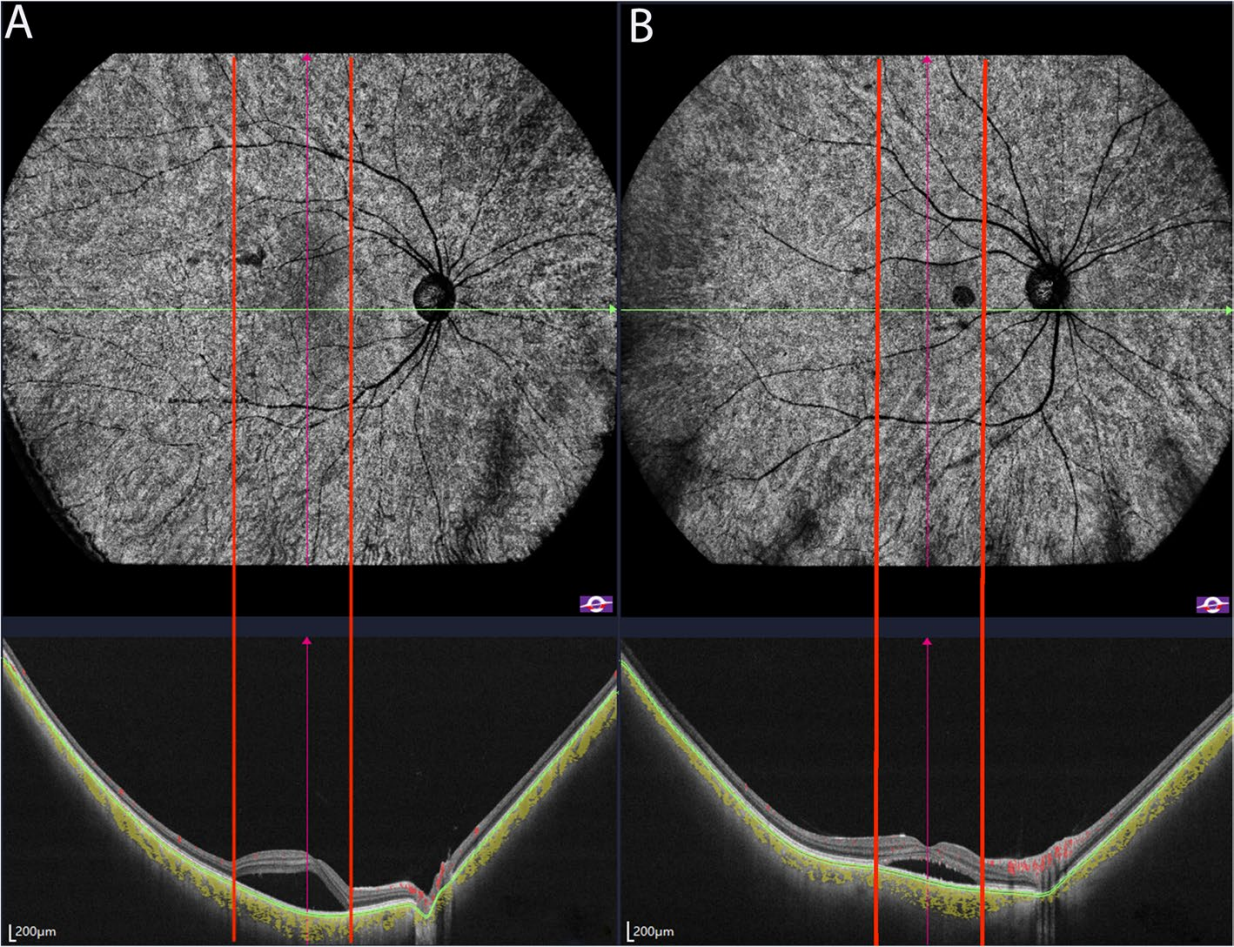
Enface choriocapillaris layer of SS-OCTA and the B-scan of the same CSC patients. Subretinal fluid (SRF) on B-scan corresponded with the area of type B abnormalities on SS-OCTA (left panel). Patients with a thinner SRF did not exhibit the corresponding halo on OCTA (right panel)

Similar to our previous study [12], all type B abnormalities were associated with SRF on structural OCT (Fig. 5). It presented in 18 eyes with acute CSC and 14 eyes with chronic CSC. Their mean maximum height of SRF was greater than that of CSC without type B abnormalities (192.5 (112.5–281.5) vs 105 (50–152), *P* = 0.0385).

Type C abnormalities were found in all the 39 eyes. The type C area corresponded with the hypofluorescent area on early-phase ICGA; however, the former was larger than the latter in all eyes, with the mean areas of 0.31 (0.18–0.56) mm^2^ and 0.17 (0.07–0.33) mm^2^ (*P* < 0.001), respectively.

### Correspondence between SS-OCTA and ICGA

All 39 eyes presented with type A abnormalities. There was no significant difference between the type A area on SS-OCTA and hyperfluorescence on ICGA (grader 1: 3.6 (IQR, 2.0–10.3) mm^2^ vs 3.4 (IQR, 2.1–8.6) mm^2^, *P* = 0.11; grader 2: 4.2 (IQR, 2.0–11.2) mm^2^ vs 4.3 (IQR, 2.2, 9.1) mm^2^, *P* = 0.21). The mean areas of type A abnormalities in acute and chronic CSC on OCTA and ICGA for the two graders were shown in Supplementary Table 1, with no significant differences.

The mean JIs, indicating the interobserver agreement of the type A abnormality on SS-OCTA and the hyperfluorescence area on ICGA, were 0.69 ± 0.11 and 0.77 ± 0.07, respectively. While the mean JI of type A abnormality on SS-OCTA and the hyperfluorescence area on ICGA was 0.55 ± 0.15 for grader 1 and 0.49 ± 0.15 for grader 2 (Table 2).

**Table 2:** Comparison of type A abnormality on SS-OCTA and hyperfluorescent area on ICGA

Parameters	Grader 1	Grader 2	Mean JIs
Hyper-fluorescence on ICGA (mm^2^)	3.43 (2.12–8.61)	4.31 (2.20–9.06)	0.77 ± 0.07
Type A on OCTA (mm^2^)	3.60 (2.00–10.31)	4.23 (1.98–11.15)	0.69 ± 0.11
Mean JIs	0.55 ± 0.15	0.49 ± 0.15	

*SS-OCTA* Swept-source optical coherence tomography angiography, *ICGA* Indocyanine green angiogram, *JI* Jaccard index

### SS-OCTA findings of fellow eyes

**Fig. 6:**
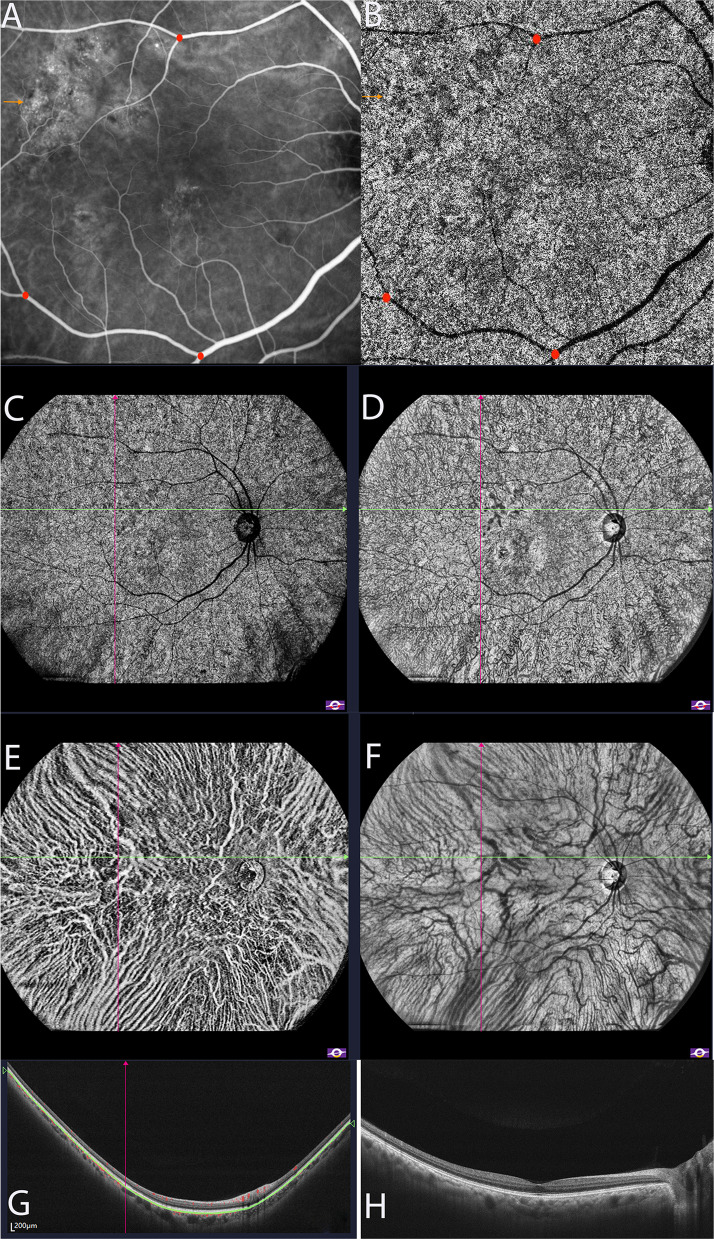
Dilated choroidal vessels of the contralateral eye identified on ICGA and SS-OCTA images. Mid-phase ICGA and choriocapillaris layer of SS-OCTA **A**, **B**. Corresponding area of hyperreflection on choriocapillaris layer of SS-OCTA **C**, en-face structural OCT **D** and B-scan **G**. Corresponding area of dilated choroidal vessels on choroidal vessel layer of SS-OCTA **E**, en-face structural OCT **F** and B-scan **G**. B-scan of the fovea structure in the contralateral eye **H**

There were 29 (85.3%) patients with unilateral eye involvement. Twenty-seven (93.1%) of them had dilated vessels on ICGA in the fellow eyes, and there were 19 (70.4%) eyes with dilated choroidal vessels and hyperreflective areas identified on SS-OCTA (Fig. 6). The mean area of type A abnormalities on SS-OCTA and hyperfluorescence on ICGA was 3.976 (IQR, 2.139–8.168) and 3.043 (IQR, 1.408–4.909) mm^2^ (*P* = 0.199).

### Changes in SS-OCTA abnormalities during the follow-up

**Fig. 7:**
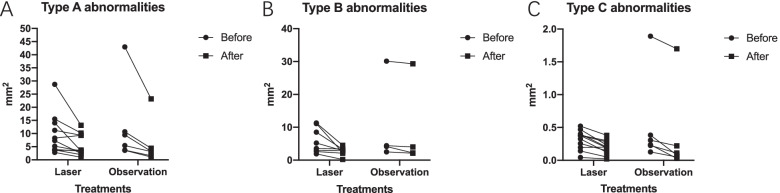
Change of SS-OCTA abnormalities between the laser and observation group during the follow-up

**Fig. 8:**
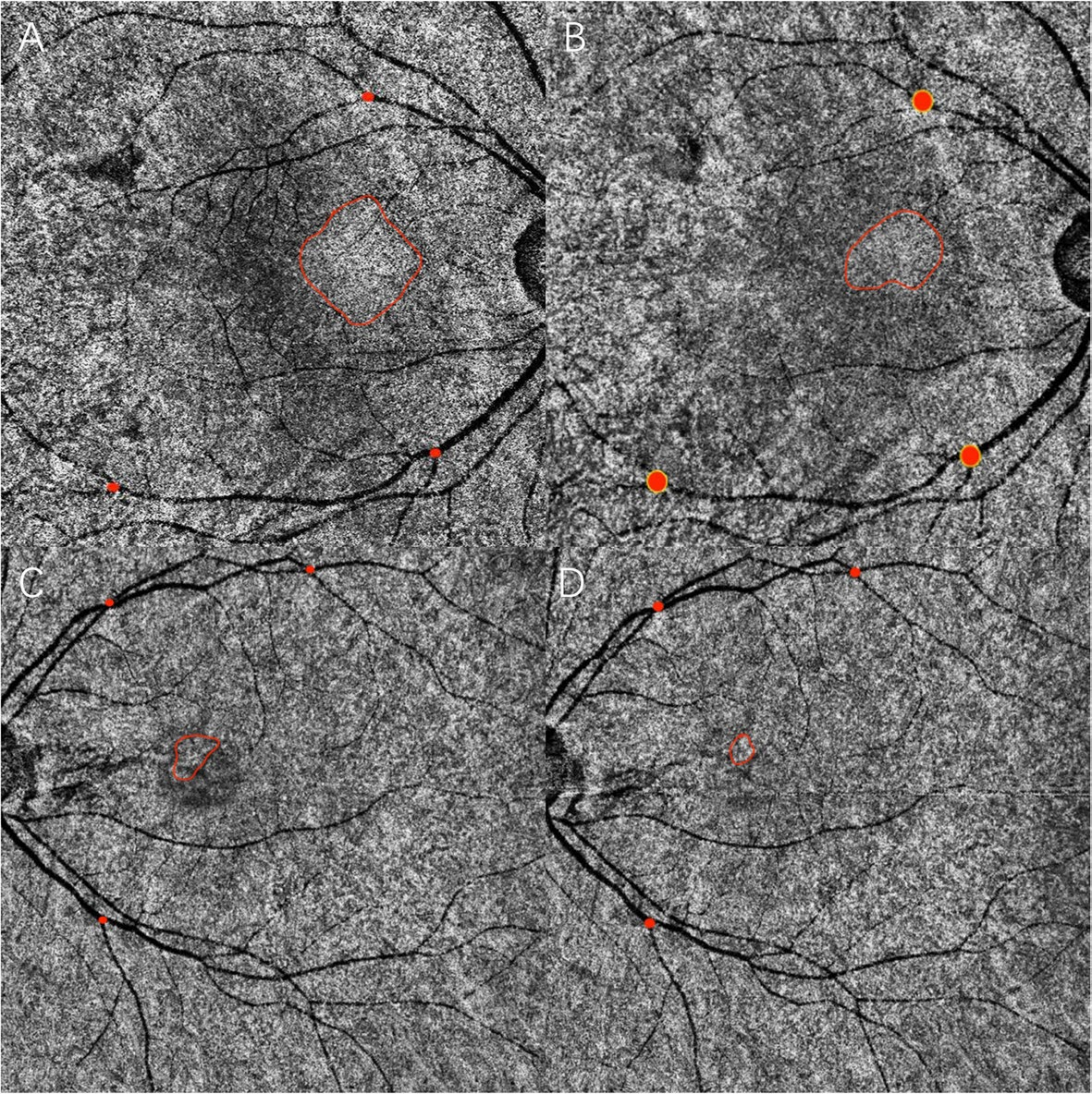
Changes in type A abnormality over time after therapy (**A-B**: 2 months after laser treatment) or observation (**C-D**: 2 months after observation)

After initial treatment, the areas of type A, B and C abnormalities on SS-OCTA were calculated during the follow-up. Nineteen (48.7%) and twenty (51.3%) eyes received observation and laser, respectively. There were 17 patients (12 with A + B + C and 5 with A + C) with regular SS-OCTA examinations during the follow-up period. The median follow-up period was 2 (IQR, 1–3) months. The areas of type A, B and C abnormalities on SS-OCTA after laser treatment or observation were 3.36mm^2^ (IQR, 2.399–9.312), 2.9mm^2^ (IQR, 2.15–3.7), and 0.19mm^2^ (IQR, 0.08–0.23), respectively, which was smaller than those in the baseline (7.311mm^2^ (IQR 3.788–11.209), *P* < 0.001; 4.3mm^2^ (IQR, 2.8–9.8), *P* = 0.002;0.33mm^2^ (IQR, 0.23–0.38), *P* < 0.001). Changes in the type A, B or C area were not significantly different between the two groups, as shown in Fig. 7 (*P* = 0.679, 0.732 and 0.892). The representative changes in type A abnormality over 2 months after therapy or observation were shown in Fig. 8.

## Discussion

The pathophysiology of CSC remains unclear. It was assumed that pachychoroid vasculature could compress the choroidal capillaries and result in dysfunction and ischemia, compensatory hyperpermeability and leakage of the around choroidal capillaries, subsequently lead to RPE dysfunction, which causes accumulation of fluid under the neuroretina [4, 13–19].

OCTA is an advanced imaging technology that enables imaging, segmentations and quantifications of blood flow in the retina and choroid. Few studies focused on comparisons between ICGA and OCTA imaging of CSC. Teussink et al. compared OCTA and ICGA in 18 eyes with chronic CSC [12]. They found reduced flow surrounded by hyperperfused areas on choriocapillaris OCTA, corresponding to ICGA abnormalities. In the study by Shinojima et al., abnormal hypofluorescence on ICGA in the late phase was detected in 25 CSC patients, corresponding to abnormal hyperreflective areas from BM to the choriocapillaris level in en face images [20]. In our previous study by Hu et al., ICGA and OCTA imaging were compared in 66 eyes [12]. In this study, three types of abnormalities in the choriocapillaris layer on SS-OCTA were found, consistent with our previous research [12]. Type A (the coarse granulated high reflective area) and C (the coarse granulated low reflective spot) abnormalities were the most commonly found in all 39 eyes.

Similar to the study by Chan et al. [21], they found that all 26 eyes showed an image pattern of high signal intensity. Gong et al. reported that 47/54 eyes with CSC presented dilated vessels on ICGA, and the corresponding areas could be recognized on OCTA [22]. In our previous study, type A was also found in most of the eyes [12]. It was assumed that type A abnormalities are relates to the dilation and increased blood flow of the choriocapillaris in CSC patients. RPE atrophy may also contribute to the type A abnormality in chronic CSC eyes; however, we found no significant difference in the type A area between acute and chronic patients, which accords closely with our previous study [12]. Type A areas could be the artifacts caused by RPE atrophy due to long-lasting SRF; nevertheless, automatic segmentation error might also cause artifacts and affect the abnormal findings at the choriocapillaris level.

There was no significant difference between the type A area on SS-OCTA and hyperfluorescence on ICGA. In the study by Hu et al. [12], 49 eyes (74.2%) exhibited an equal area of type A abnormality on OCTA and a hyperfluorescent area on ICGA, similar to our findings. In the study by Teussink et al. [23], chronic CSC also showed irregular choriocapillary flow patterns corresponding to ICGA abnormalities.

The interobserver agreement of type A abnormality on SS-OCTA and the hyperfluorescence area on ICGA was high. There was moderate spatial correspondence between type A abnormality on SS-OCTA and hyperfluorescence area on ICGA, with a mean JI of 0.55 ± 0.15 for grader 1 and 0.49 ± 0.15 for grader 2. The previous study by Hu et al. from our team indicated a higher JI of the abnormality area between the two modalities [12]. They applied a circumcircle of the abnormal area instead of the border of the abnormal area on OCTA to calculate the JIs, which may result in a larger overlapping area than they actually were. The different technologies of OCTA could also cause the heterogeneity of the results. Moreover, 6 × 6 mm en face choriocapillary OCT images in the previous study cover a smaller area than the cropped en face SS-OCT images. Our present research is an improvement and supplement to the original study by Hu et al. [12].

Type C abnormalities were discovered in all the eyes in our study, consistent with the study by Shinojima et al. and Kitaya et al. Type C abnormalities could be the thinned choriocapillaries pushed upward by pachyvessls or low blood flow at the choriocapillary level [24–26]. We also revealed that the type C abnormal area corresponded with the hypo-fluorescence area on early-phase ICGA, with the former larger than the latter. We postulated that in ICGA, leakage of choriocapillaris in the early-phase, or staining from subretinal fibrin content in the later phase could block the hypo-fluorescence area [23, 27]. OCTA might be more valuable than ICGA in revealing choriocapillary hypoperfusion.

Patients with the type B abnormality had a greater mean maximum SRF thickness than patients without type B abnormalities. We assumed that the type B abnormality was the shadow effect caused by SRF. We must be cautious in interpreting shadowing effects, not only due to pigment epithelial detachment but also to elongation or irregularities of the photoreceptor outer segment or other artifacts [25, 28, 29].

Fellow eyes of CSC without definite RPE alterations might also have abnormal choriocapillaris perfusion as a consequence of underlying choroidal vessels [30]. In the study by Gong et al., 13 of 21 (61.9%) contralateral eyes without SRD or leakage presented dilated vessels on ICGA and were recognized on OCTA [22]. We should pay attention to the abnormalities on OCTA of fellow eyes in the clinic.

Laser photocoagulation has been attempted to “seal” the RPE leakage points. The discovery of choriocapillary changes following laser in this study provides new evidence to support these hypotheses. Resolution of SRF corresponded well with the statistically decreased area of type B abnormality during the follow-up. Most likely due to limited cases with regular follow-up, we found no difference between the laser and observation groups. Most of the 17 patients had acute CSC, which may also explain for the indifference. It was indicated that SS-OCTA may become a useful tool in the follow-up of CSC. Larger studies with longer follow-up periods are needed to quantify the change in abnormalities on SS-OCTA in CSC patients after diverse treatments.

Among existing OCTA studies of CSC, we have a relatively large sample size, and we investigated the fellow eyes as well as changes in abnormalities during the follow-up. The SS-OCTA device we applied displayed the deeper penetration and higher resolution than those of published studies. However, there are also several limitations in our study. First, its retrospective nature cannot be neglected. Second, larger prospective and longitudinal series with enough time of follow-up time are needed to further confirm our results. Third, the semiautomatic segmentation and annotation methods could also introduce bias. Fourth, only taking the choriocapillaris into account in SS-OCTA may limit the scope of our results.

## Conclusions

In conclusion, SS-OCTA promotes noninvasive visualization and follow-up quantifications of the choroidal vasculature in CSC patients. Coarse granulated low reflective area and coarse granulated high reflective area on choriocapillaris OCTA were the most common abnormalities, providing evidence for choriocapillary ischemia and compensatory dilation in the pathogenesis of CSC. Coarse granulated high reflective area in SS-OCTA corresponded well with the hyperpermeability area in ICGA. More larger studies are needed to quantify the change in abnormalities on SS-OCTA in CSC patients after treatments.

## Supplementary Information

**Additional file 1: Supplementary Table 1.** The mean areas of type A abnormalities in acute and chronic CSC on SS-OCTA and ICGA for the two graders.

## Data Availability

The datasets used and/or analyzed during the current study are available from the corresponding author on reasonable request.

## Abbreviations

CSC: Central serous chorioretinopathy
RPE: Retinal pigment epithelium
FA: Fluorescein angiography
SRF: Subretinal fluid
ICGA: Indocyanine green angiogram
OCTA: Optical coherence tomography angiography
SS-OCTA: Swept-source optical coherence tomography angiography
CNV: Choroidal neovascularization
PCV: Polypoidal choroidal vasculopathy
DR: Diabetic retinopathy
RVO: Retinal vein occlusion
BCVA: Best corrected distance visual acuity
IOP: Intraocular pressure
AL: Axial length
HMADA: Higher-order moments amplitude decorrelation angiography
AI: Artificial intelligence
BM: Bruch’s membrane
ROI: Region-of-interest
JI: Jaccard index
SD: Standard deviation
IQR: Interquartile range
ANOVA: Two-way analysis of variance
logMAR: Logarithmic minimum angle of resolution
