# Supplementary material for: Comparison of swept-source OCTA and indocyanine green angiography in central serous chorioretinopathy
Source: BMC Ophthalmol. 2022 Sep 22;22:380. doi: 10.1186/s12886-022-02607-4 (PMC9502960; doi:10.1186/s12886-022-02607-4)
Supplement: Supplementary file 1 — Additional file 1: Supplementary Table 1. The mean areas of type A abnormalities in acute and chronic CSC on SS-OCTA and ICGA for the two graders. [file 12886_2022_2607_MOESM1_ESM.docx]

Supplementary Table 1 The mean areas of type A abnormalities in acute and chronic CSC on SS-OCTA and ICGA for the two graders

|  | CSC | SS-OCTA | ICGA | P-value |
| --- | --- | --- | --- | --- |
| Grade 1 | Acute | 3.4 (2.2, 6.8) | 3.3 (2.1, 7.1) | 0.223 |
| (mm^2^) | Chronic | 5.0 (1.5, 10.7) | 5.6 (2.4, 9.7) | 0.234 |
|  | P-value | 0.932 | 0.734 | NA |
| Grade 2 | Acute | 4.2 (2.6, 7.4) | 3.4 (2.2, 7.4) | 0.339 |
| (mm^2^) | Chronic | 5.5 (1.7, 12.2) | 6.0 (2.3, 9.9) | 0.535 |
|  | P-value | 0.932 | 0.734 | NA |

SS-OCTA, swept-source optical coherence tomography angiography; ICGA, indocyanine green angiogram; CSC, central serous chorioretinopathy; NA, not applicable.
